# Separate foliar sodium selenate and zinc oxide application enhances Se but not Zn accumulation in pea (*Pisum sativum* L.) seeds

**DOI:** 10.3389/fpls.2022.968324

**Published:** 2022-11-01

**Authors:** Maksymilian Malka, Gijs Du Laing, Jun Li, Torsten Bohn

**Affiliations:** ^1^ Laboratory of Analytical Chemistry and Applied Ecochemistry, Department of Green Chemistry and Technology, Faculty of Bioscience Engineering, Ghent University, Ghent, Belgium; ^2^ Nutrition and Health Research Group, Department of Precision Health, Luxembourg Institute of Health, Strassen, Luxembourg

**Keywords:** legume biofortification, selenate, zinc oxide, mineral deficiency, nutrition, food security, HPLC-ICP/MS

## Abstract

Up to 15% and 17% of the world population is selenium (Se) and zinc (Zn) deficient, respectively. Pea (*Pisum sativum* L.) is an important staple legume with a high potential for Se and Zn biofortification in seeds. A 2-year pot experiment investigated two pea varieties (Ambassador and Premium) following foliar-applied sodium selenate (0/50/100 g of Se/ha) and zinc oxide (0/375/750 g of Zn/ha) at the flowering stage. Selenate and zinc oxide had minimal overall effects on growth parameters. Zinc oxide did not improve Zn accumulation in both seed varieties, while selenate improved Se accumulation in both seed varieties dose-dependently. Premium accumulated greater amounts of Se in seeds than Ambassador (*p* < 0.001). Selenium concentrations were highest in seeds of Premium treated with 100 g of Se/ha [7.84 mg/kg DW vs. the control (0.16 mg/kg DW), *p* < 0.001]. The predominant Se species in Se-enriched seeds was selenomethionine (40%–76% of total Se). Furthermore, a significant (*p* < 0.01) positive correlation was found between Zn and S concentrations in Ambassador (*r* ^2^ = 0.446) and Premium (*r* ^2^ = 0.498) seeds, but not between Se and S. Consuming as little as 55 g/day of pea biofortified by 50 g of Se/ha would cover 100% of the adult RDA (55 µg) for Se. Findings are important for improving foliar biofortification of pea with Se and Zn.

## Introduction

Selenium (Se) and zinc (Zn) are essential trace elements for humans. Selenium (as selenoproteins) and Zn are involved, *via* their role as enzymatic co-factors, in a large number of antioxidant defense and immune functions, such as being co-factors for glutathione peroxidase and superoxide dismutase, respectively (Marreiro et al., 2017; Barchielli et al., 2022). A limited intake or low circulating concentrations of Se and Zn have been associated with increased risk of mortality and several non-communicable chronic diseases. These include cancer, neurodegenerative diseases, and cardio-metabolic complications, such as type 2 diabetes or metabolic syndrome (Rayman, 2012; Kaur et al., 2014; Barchielli et al., 2022). A relative lack of Se and Zn has also been associated with infectious diseases, including COVID-19, likely due to their participation in antioxidant and anti-inflammatory and thus immune-relevant processes in the body (Alexander et al., 2020; Du Laing et al., 2021). However, excessive intakes of both Se and Zn may also cause health problems, as reviewed previously (Rayman, 2020; Agnew & Slesinger, 2022).

According to some sources, it is estimated that up to 15% and 17% of the world population is Se and Zn deficient, respectively (Kumssa et al., 2015; Shreenath et al., 2022). Suboptimal Se and Zn statuses were reported to be also widespread throughout Europe. This reflects, to a large extent, inadequate soil levels (Tóth et al., 2016), as they have become depleted by agricultural use and rainfall. Main dietary sources for Se include cereals and grains, while for Zn, meat and meat products are the prominent source (Olza et al., 2017), though cereals and grains are the second predominant source. Therefore, intakes of Se and Zn depend largely on their concentrations in soil and the bioavailability from major crops (Alloway, 2008; Chilimba et al., 2011; Winkel et al., 2015). However, soil Se and Zn are uneven in their distribution and availability to plants, as reviewed earlier (Sadeghzadeh, 2013; Jones et al., 2017).

Biofortification is a promising agricultural strategy to improve the level of micronutrients in staple foods. This strategy encompasses classical plant breeding, genetic engineering, and agronomic biofortification. The latter is based on optimized fertilizer application to the soil and/or crop leaves in the case of foliar biofortification, as reviewed previously (de Valença et al., 2017; Cakmak and Kutman, 2018; Szerement et al., 2022). It has been shown that foliar spraying is a highly effective method of plant biofortification for Se and Zn (Delaqua et al., 2021; Sattar et al., 2021). The efficiency of foliar applied trace elements is affected by numerous factors. These include physicochemical properties of the formulation, the environmental conditions under which spraying is carried out, or the characteristics of the plant to which spraying is applied, as reviewed previously (Fernández and Brown, 2013).

Legumes constitute staple foods for billions of people around the world. However, legume biofortification has been emphasized as an underexploited strategy for combatting hidden hunger (Rehman et al., 2018; Kumar and Pandey, 2020). Pea (*Pisum sativum* L.) is an important legume crop produced worldwide and employed for animal and human nutrition. In 2020, the world production of dry peas amounted to 14.6 million tons, with cultivated areas covering 7.2 million hectares (FAOSTAT 2022). Pulses (including peas) are beneficial for sustainable agriculture and environment, biodiversity, global health, and food security (Powers & Thavarajah, 2019; Ferreira et al., 2021). These crops are of high nutritional value, play an essential role in cropping systems, enhance soil health, and reduce synthetic nitrogen fertilizer applications and associated fossil energy consumption (Sahruzaini et al., 2020; Ferreira et al., 2021). Peas are a good and affordable source of high-biological-value protein (Ge et al., 2020), complex carbohydrates, dietary fiber, starch, vitamins, minerals, and phytochemicals that may favorably affect human health (Dahl et al., 2012). The intake of peas and their constituents has been associated with metabolic, cardiovascular, and gastrointestinal health benefits (Dahl et al., 2012; Kumari and Deka, 2021). Regarding Se, its availability from crops does, to a large degree, depend not only on the total amount of Se but also on the chemical speciation of Se in the food crops. It is understood that organic Se species are absorbed more effectively and are considered less toxic at higher intakes than inorganic species (Zhang et al., 2013). However, studies on Se speciation in legumes are also limited (Smrkolj et al., 2006; Poblaciones et al., 2014; Thavarajah et al., 2015).

The aim of this 2-year pot experiment was to examine the effect of foliar-applied Se (sodium selenate) and Zn (zinc oxide) at the flowering stage on two pea varieties (Ambassador and Premium). The experiment consisted of five treatments, including one un-amended control and two levels of applications for both Se and Zn. Growth parameters; Se, Zn, and sulfur (S) concentrations (due to potential interactions with Se); and Se speciation were determined in seeds. To the best of our knowledge, the present investigation is only the second study to investigate Se speciation following foliar biofortification of peas (Smrkolj et al., 2006).

## Materials and methods

### Chemicals

Zinkuran SC was purchased from Arysta LifeScience Slovakia s.r.o. (Nové Zámky, Slovakia). Sodium selenate was obtained from Alfa Aesar (Karlsruhe, Germany). Nitric acid (HNO_3_, for trace element analysis) and hydrogen peroxide 30% (Suprapur) were acquired from LGC Standards (Molsheim, France) and Merck/VWR (Leuven, Belgium), respectively. Sodium selenite (Na_2_SeO_3_), sodium selenate (Na_2_SeO_4_), Se-methionine (SeMet), Se-cystine (SeCys_2_), and Se-methyl-selenocysteine (SeMetSeCys) were purchased from Sigma Aldrich (St. Louis, MO, USA). Protease XIV, citric acid, and methanol were from Sigma Aldrich. MilliQ (MQ) water from Water Systems Ltd. (Brussels, Belgium) was used throughout the experiment.

### Design of experiment and sample preparation

A 2-year outdoor pot experiment, during which plants were not fully exposed to outdoor conditions, was conducted in 2014 and 2015 in the Botanical Garden of the Slovak University of Agriculture in Nitra (48.305 N, 18.096 E), Slovakia. The experiment was arranged with four replicates per treatment, two pea varieties, and five different treatments (total of 80 pots over two growing seasons). The average monthly air temperature and total monthly rainfall in the 2014 growing season were as follows: March (9.3°C and 15.4 mm), April (12.4°C and 48.9 mm), May (15.2°C and 57.6 mm), and June (19.3°C and 52.5 mm), while in the 2015 growing season, the corresponding values were as follows: March (6.3°C and 35.4 mm), April (10.4°C and 25.0 mm), May (15.1°C and 69.5 mm), and June (19.9°C and 10.2 mm). A gleyic fluvisol soil (that is the typical soil type of the area) was employed in the experiment. The soil from the 2014 growing season had a pH of 6.47 and contained 19.5 mg kg^−1^ of N, 86.3 mg kg^−1^ of P, 498 mg kg^−1^ of K, 6,610 mg kg^−1^ of Ca, 816 mg kg^−1^ of Mg, 26.3 mg kg^−1^ of S, 2.47 mg kg^−1^ of Zn, 0.08 mg kg^−1^ of Se, and 3.46% of humus. The soil from the 2015 growing season had a pH of 7.16 and contained 19.1 mg kg^−1^ of N, 245 mg kg^−1^ of P, 150 mg kg^−1^ of K, 6,340 mg kg^−1^ of Ca, 644 mg kg^−1^ of Mg, 7.5 mg kg^−1^ of S, 2.39 mg kg^−1^ of Zn, 0.08 mg kg^−1^ of Se, and 3.25% of humus. Soil was collected with a soil corer with a sampling depth of 0–0.3 m. The concentration of elements in soils was determined according to the method of Varényiová et al. (2017) for total N and S, and available P, K, Mg, and Ca; Ducsay et al. (2009) for total Se; and Lindsay and Norvell (1978) for available Zn.

Two pea varieties, i.e., Ambassador (late variety, restored hybrid) and Premium (early variety, open pollinated), were selected for the experiments. Seeds were purchased from a local farmer. Ten-liter plastic square pots were filled with soil and placed in a wire mesh housing to protect plants against bird attacks. Thirty seeds/pot were sown in two rows at 5 cm depth in mid-March. Selenium as sodium selenate and Zn as Zinkuran SC (30% ZnO + 6% chelate) were applied in the experiment. The experiment consisted of five treatments: un-amended control (control), 50 g of Se/ha (Se1), 100 g of Se/ha (Se2), 375 g of Zn/ha (Zn1), and 750 g of Zn/ha (Zn2). The solutions employed contained 0.1 and 0.2 g/L of Se and 0.75 and 1.5 g/L of Zn. Foliar applications of Se and Zn were performed at the flowering stage of plants during non-rainy periods. A plastic trigger spray bottle was used for the manual application of fertilizers. No additional fertilization was employed. Watering and weed and snail removal were carried out regularly. Toxic effects of foliar Se and Zn treatments on plants or incidences of pests and diseases were not observed during the experiment. Freshly harvested seeds were immediately lyophilized, homogenized by grinding, and the concentrations of Se, Zn, and S, and Se species were examined.

### Growth parameters

Number of seeds per pod, pod length, and pod perimeter were measured after harvest. Samples were dried at 105°C in a drying oven to a constant weight for seed dry matter determination.

### Concentrations of total Se, Zn and S in seeds

An aliquot (0.2 g) of each sample was mixed with 3.5 ml of HNO_3_ (65%) and 3.5 ml of H_2_O_2_ (30%). Thereafter, microwave digestion for complete combustion of organic matrix was carried out using a MARS 6 system (CEM, Orsay Cedex, France, 1,200 W, 10 min at 55°C, 10 min at 75°C, and 45 min at 120°C). Total Se, Zn, and S concentrations [mg/kg dry weight (DW)] were subsequently determined in the diluted digests *via* an inductively coupled plasma mass spectrometer (ICP-MS, PerkinElmer Elan DRCe, Waltham, MA, USA, for Se) and an inductively coupled plasma optical emission spectrometer (ICP-OES, Varian Vista MPX, Palo Alto, CA, USA, for Zn and S), respectively. External calibration was used. Accuracy and precision were monitored by periodic evaluation of a calibration blank, re-analyzing standards during sample runs, and analysis of certified reference materials (including rice flour NIST1568a, sea lettuce BCR279 and spinach leaves SRM 1570a), spiked samples and analytical duplicates. Analytical batches were rejected and reanalysis was planned when the concentrations measured in reanalyzed standards, certified reference materials, or spiked samples deviated more than 10% from the expected/certified value.

### Selenium speciation in seeds

Selenium speciation analysis was determined according to Lavu et al. (2013); Lavu et al. (2012). The seeds of pea (Ambassador and Premium variety) treated with 100 g of Se/ha as selenate were selected for Se speciation analysis. Specifically, 0.2 g of whole plant samples and 80 mg of the enzyme protease XIV were dispersed in 5 ml of water in a 10-ml centrifuge tube. The mixture was shaken for 24 h at 37°C and centrifuged for 30 min at 10,000 *g*. The supernatant was filtered through a 0.25-µm syringe PVDF membrane filter. The filtrate was analyzed for Se speciation by an ICP-MS (PerkinElmer DRC-e, Sunnyvale, CA, USA) coupled to a high-performance liquid chromatograph (Series 200 HPLC, Perkin Elmer, Sunnyvale, CA, USA), respectively. A Hamilton PRP-X100 anion exchange column (250 mm × 4.6 mm, 5 μm) was used as stationary phase in the HPLC instrument. The isocratic mobile phase was 10 mM citric acid with 5% (v/v) methanol, adjusted to pH 5.0. The standard solutions of the different Se species were prepared with sodium selenite (Na_2_SeO_3_), sodium selenate (Na_2_SeO_4_), Se-methionine (SeMet), Se-cystine (SeCys_2_), and Se-methyl-selenocysteine (SeMetSeCys).

### Statistical analysis

Normal distribution of data and equality of variance were verified by normality plots and box plots, respectively. Whenever required, data were log-transformed in order to achieve normal distribution. Multivariate models were then employed, with seed dry matter, number of seeds per pod, pod perimeter, pod length, and Se, Zn, and S concentrations in seeds as the observed (dependent) variables, and genetic variant (two levels), year (two levels), and biofortificant type (five levels, two for Se, two for Zn, and controls) as independent, fixed factors. Biofortification levels were nested within biofortificant. Following significant Fisher *F*-tests, all group-wise comparisons were carried out (Bonferroni post-hoc tests). In case of significant interactions, models were re-run with one of the significant interacting terms kept constant. A *p*-value <0.05 (two-sided) was considered statistically significant. SPSS, version 25.0 (IBM, Chicago, IL, USA), was used for all analyses including Pearson correlation analyses.

## Results

### Growth parameters

Following multivariate models, combined analysis of variance showed that treatment (pooled years and varieties) significantly affected all examined variables except for the number of seeds per pod. Growing year (pooled treatments and varieties) had a significant effect on all variables except for pod length. Variety (pooled treatments and years) showed a significant effect on all variables. Interactions were significant in some cases (**Table 1**).

**Table 1:** Combined analysis of variance for the effects of year, variety, and treatment on seed dry matter, number of seeds per pod, pod length, pod perimeter, and seed Se, Zn, and S concentrations.

	DF	Seed dry matter (%)	Number of seeds/pod	Pod length(cm)	Pod perimeter (cm)	Se (mg/kg DW)	Zn (mg/kg DW)	S (mg/kg DW)
Year (Y)	1	<0.001	<0.001	NS	<0.001	NS	<0.001	0.002
Variety (V)	1	<0.001	<0.001	<0.001	<0.001	0.003	<0.001	NS
Treatment (T)	4	<0.001	NS	0.049	0.025	<0.001	NS	NS
Y × V	1	0.001	<0.001	<0.001	0.044	0.020	0.001	0.008
Y × T	4	0.007	NS	NS	NS	0.026	0.011	NS
V × T	4	<0.001	<0.001	NS	0.012	NS	0.002	NS
Y × V × T	4	0.003	<0.001	NS	0.019	NS	NS	NS

DF, degrees of freedom; NS, not significant.

When investigating effects per year, in 2014, the Zn1 treatment significantly decreased seed dry matter of Ambassador vs. the control, while Se1, Se2, and Zn2 significantly decreased seed dry matter of Premium vs. the control. Ambassador showed significantly higher seed dry matter than Premium for the control, Se1, Zn2, and a trend for Se2 treatment. Also, Zn1 and Zn2 significantly increased the number of seeds per pod of Ambassador vs. the control. In contrast, Zn1 and Zn2 significantly decreased the number of seeds per pod of Premium vs. the control. Premium showed a significantly higher number of seeds per pod than Ambassador for Se2. Ambassador showed a significantly higher number of seeds per pod than Premium for Zn1 and Zn2. In 2014, treatment did not significantly influence pod length of Ambassador vs. the control, while it had a marginal significant effect on the pod length of Premium vs. the control (though individual group-wise comparison with post-hoc correction did not reveal differences due to correction for multiple comparison). Ambassador showed a significantly higher pod length than Premium for all treatments. Finally, in 2014, treatment did not significantly affect pod perimeter of Ambassador vs. the control. Pod perimeter of Premium was significantly increased by Se1 and Se2 vs. Zn1 and Zn2. Premium showed significantly higher pod perimeter than Ambassador for Se2.

In 2015, the Zn1 treatment significantly decreased seed dry matter of Ambassador vs. the control, while the Se2 treatment significantly increased seed dry matter of Premium vs. the Zn2 treatment. Ambassador showed significantly higher seed dry matter than Premium for the control, Se1, and Zn2. Furthermore, for 2015, treatment did not significantly affect the number of seeds per pod of both varieties vs. controls. No significant differences were found for the number of seeds per pod between Ambassador and Premium for all treatments. Treatment did not significantly affect pod length of both varieties vs. controls. Ambassador showed significantly higher pod length than Premium for Se2 and Zn1. Finally, in 2015, treatment did not significantly influence pod perimeter of both varieties vs. controls. Ambassador showed significantly higher pod perimeter than Premium for Se1, Se2, Zn1, and Zn2.

Comparing growing year per variety, it significantly affected seed dry matter and the number of seeds per pod of both varieties, it had a significant effect on the pod perimeter of Premium (not Ambassador), though it did not significantly affect pod length of both varieties (**Table 2**).

**Table 2:** Effect of foliar-applied Se and Zn, variety, and year on seed dry matter, number of seeds per pod, pod length, and pod perimeter.

		Seed dry matter (%)			Number of seeds per pod			Pod length (cm)			Pod perimeter (cm)		
Year	Treatment	Ambassador	Premium	*p*-value	Ambassador	Premium	*p*-value	Ambassador	Premium	*p*-value	Ambassador	Premium	*p*-value
2014	Control	28.5 ± 1.19^B^	24.6 ± 0.38^C^	**<0.001**	5.20 ± 0.98^A^	5.08 ± 0.85^B^	0.863	6.54 ± 0.18	5.57 ± 0.27^A^	**<0.001**	4.13 ± 0.13	4.07 ± 0.09^AB^	0.542
	Se1	26.6 ± 0.34^B^	23.6 ± 0.16^B^	**<0.001**	4.62 ± 0.23^A^	4.95 ± 0.18^B^	0.062	6.82 ± 0.20	5.57 ± 0.14^A^	**<0.001**	4.21 ± 0.12	4.26 ± 0.10^B^	0.509
	Se2	26.0 ± 1.40^B^	24.2 ± 0.54^B^	0.053	4.35 ± 0.25^A^	5.00 ± 0.05^B^	**0.002**	6.72 ± 0.23	5.55 ± 0.13^A^	**<0.001**	4.09 ± 0.05	4.31 ± 0.01^B^	**<0.001**
	Zn1	21.1 ± 4.98^A^	23.6 ± 0.17^BC^	0.343	6.55 ± 0.62^B^	3.50 ± 0.58^A^	**<0.001**	6.36 ± 0.64	5.15 ± 0.24^A^	**0.012**	4.19 ± 0.35	3.88 ± 0.14^A^	0.143
	Zn2	27.9 ± 0.79^B^	19.3 ± 0.28^A^	**<0.001**	6.50 ± 0.49^B^	3.88 ± 0.57^A^	**<0.001**	6.53 ± 0.50	5.23 ± 0.33^A^	**0.005**	4.19 ± 0.10	3.81 ± 0.33^A^	0.073
	*p*-value	**<0.005**	**<0.001**		**<0.001**	**0.001**		0.524	**0.046**		0.869	**0.003**	
2015	Control	22.3 ± 0.98^B^	20.9 ± 0.55^AB^	**0.049**	4.40 ± 0.89	4.27 ± 0.54	0.812	6.14 ± 0.45	5.70 ± 0.46	0.223	3.44 ± 0.23	3.44 ± 0.02	0.980
	Se1	22.5 ± 0.29^B^	21.1 ± 0.86^AB^	**0.018**	4.08 ± 0.39	4.29 ± 0.26	0.414	6.17 ± 0.33	5.63 ± 0.37	0.073	3.59 ± 0.18	3.32 ± 0.05	**0.024**
	Se2	21.9 ± 0.79^AB^	22.1 ± 0.48^B^	0.688	4.29 ± 0.54	4.03 ± 0.44	0.471	6.24 ± 0.35	5.66 ± 0.32	**0.048**	3.76 ± 0.10	3.45 ± 0.09	**0.004**
	Zn1	20.6 ± 1.02^A^	21.3 ± 1.14^AB^	0.385	3.92 ± 0.83	3.83 ± 0.18	0.824	6.03 ± 0.33	5.29 ± 0.30	**0.015**	3.63 ± 0.13	3.30 ± 0.15	**0.016**
	Zn2	22.6 ± 0.54^B^	20.2 ± 0.03^A^	**<0.001**	4.28 ± 0.26	4.63 ± 0.76	0.422	6.35 ± 0.32	5.68 ± 0.49	0.065	3.64 ± 0.11	3.39 ± 0.08	**0.010**
	*p*-value	**0.013**	**0.034**		0.833	0.231		0.777	0.564		0.131	0.102	
	^*^ *p*-value across	**<0.001**	**<0.001**		**0.003**	**0.002**		0.488	0.084		0.446	**<0.001**	

Control: without Se/Zn, Se1: 50 g of Se/ha, Se2: 100 g of Se/ha, Zn1: 375 g of Zn/ha, Zn2: 750 g of Zn/ha; mean ± SD; n = 4. Means within a column followed by different letters are significantly different. p-values in the same row mean the effect of Se/Zn dose. p-values in the same column mean the effect of variety. ^*^p-values across refer to the effect of year. p-values in bold are statistically significant.

### Se, Zn and S concentrations in seeds

Following multivariate models, combined analysis of variance showed that treatment (pooled years and varieties) significantly affected only Se concentration. Growing year (pooled treatments and varieties) had a significant effect on Zn and S concentrations. Variety (pooled treatments and years) showed a significant effect on Se and Zn concentrations. Interactions were significant in some cases (**Table 1**).

**Figure 1:**
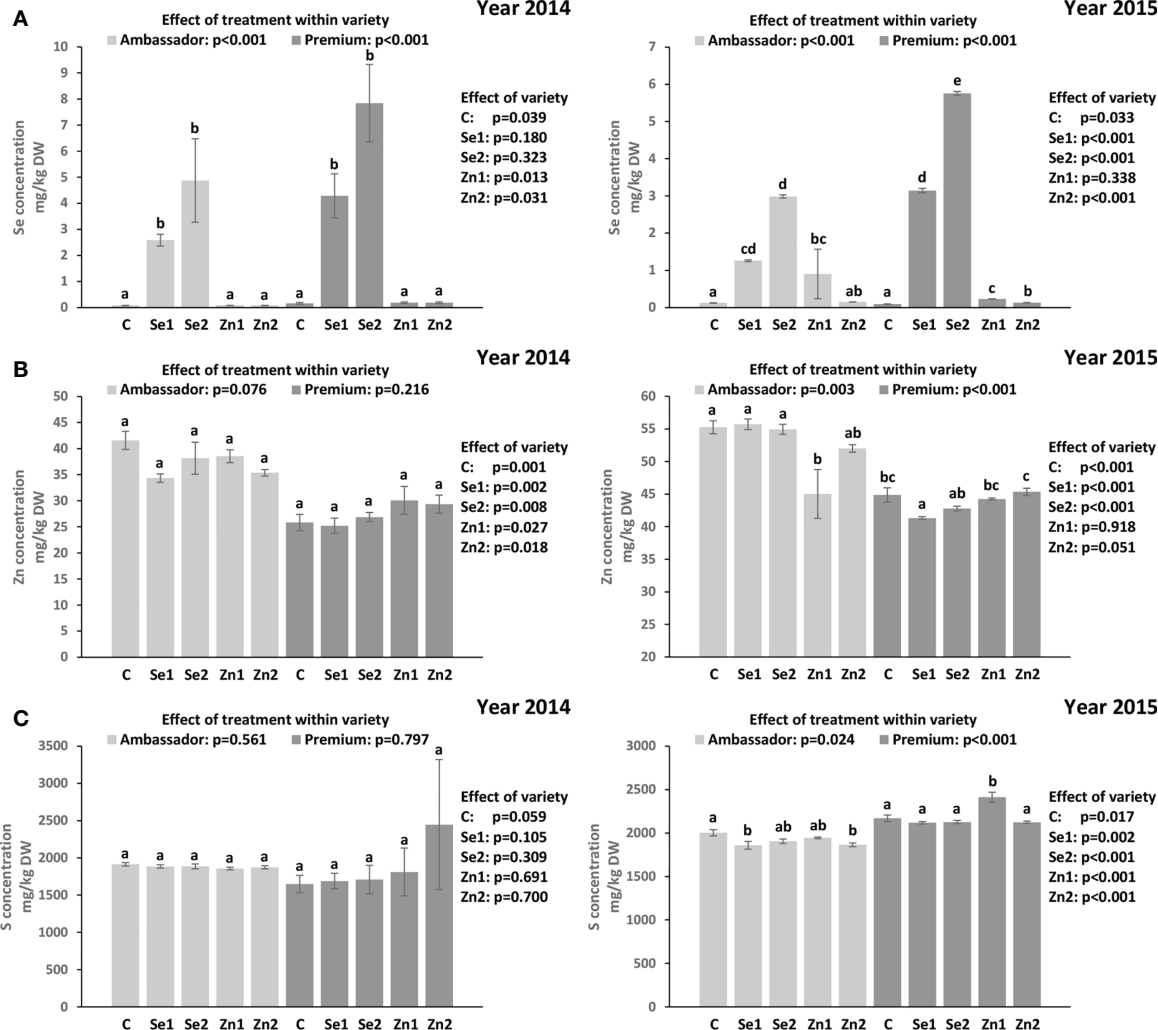
Effect of foliar Se and Zn treatments and variety in two growing seasons (2014 and 2015) on the Se concentration **(A)**, Zn concentration **(B)**, and S concentration **(C)** in pea seeds. Control: without Se/Zn; Se1: 50 g of Se/ha; Se2: 100 g of Se/ha; Zn1: 375 g of Zn/ha; Zn2: 750 g of Zn/ha; mean ± SD; *n* = 4. Bars not sharing the same superscript are significantly different within variety. *p*-values on the right side of the figure show the effect of treatment across the two varieties (i.e., Ambassador vs. Premium).

For both years, Se treatment significantly increased Se concentration vs. controls in Ambassador and Premium. In 2014, the highest Se concentration was found in Premium treated with Se2 vs. the control and in Ambassador treated with Se2 vs. the control. Also, no significant differences were observed in Se concentration between Ambassador and Premium for Se1 and Se2. Premium showed significantly higher Se concentration than Ambassador for the control. In 2015, the highest Se concentration was found in Premium treated with Se2 and Se1 vs. the control. Also, Premium showed significantly higher Se concentration than Ambassador for Se1 and Se2 in 2015. Contrarily, Ambassador showed slightly but significantly higher Se concentration than Premium for the control (**Figure 1A**). Growing year had no significant effect on Se concentration in both varieties (*p* > 0.05).

In 2014, treatment did not significantly influence Zn concentration vs. controls in both varieties. Ambassador showed significantly higher Zn concentration than Premium for all treatments. Also, treatment did not significantly affect S concentration vs. controls in both varieties. No significant differences were observed in S concentration between Ambassador and Premium for all treatments. In 2015, the Zn1 treatment significantly decreased Zn concentration vs. the control in Ambassador, while Se1 significantly decreased Zn concentration vs. the control in Premium. Ambassador showed significantly higher Zn concentration than Premium for the control, Se1, Se2, and a trend for Zn2 treatment (**Figure 1B**). Also, treatment significantly influenced S concentration vs. controls in both varieties. In Ambassador, Se1 and Zn2 decreased S concentration vs. the control, while in Premium, Zn1 increased S concentration vs. the control. Premium showed significantly higher S concentration than Ambassador for all treatments (**Figure 1C**). Growing year significantly affected Zn concentration in both varieties (*p* < 0.001), and it had a significant effect on S concentration in Premium (*p* = 0.005) compared with Ambassador (*p* = 0.075).

### Se speciation in seeds

Selenium species recovery ranged between 62% and 106% after protease hydrolysis for samples treated with 100 g of Se/ha (**Table 3**). The chromatogram of the standard solution containing all determined Se species is shown in **Figure S1C**. The predominant Se species identified in pea seeds was SeMet, ranging between 58% and 76% of total Se in Ambassador and between 40% and 71% of total Se in Premium. The species SeCys, SeMetSeCys, Na_2_SeO_3_, and Na_2_SeO_4_ were also identified, although in much lower proportions (**Table 3** and **Figure S1**).

**Table 3:** Selenium species concentrations and percentage (given in brackets) of Se species of total µg/g Se in seeds of pea grown with the foliar treatment of 100 g of Se/ha in the form of selenate.

Sample	SeCys (µg/g)	SeMetSeCys (µg/g)	Na_2_SeO_3_ (µg/g)	SeMet (µg/g)	Na_2_SeO_4_ (µg/g)	Total Se (µg/g)	Se species recovery (%)
A1	0.15 (2.35%)	0.20 (3.04%)	0.04 (0.69%)	3.73 (57.8%)	0.91 (14.0%)	6.46	78%
A2	0.26 (3.86%)	0.40 (5.89%)	0.12 (1.82%)	5.16 (76.1%)	1.27 (18.8%)	6.78	106%
A3	0.29 (4.70%)	0.77 (12.5%)	ND	4.11 (66.6%)	1.29 (20.9%)	6.16	105%
B1	0.17 (2.96%)	0.06 (0.97%)	0.09 (1.52%)	2.30 (40.1%)	0.93 (16.1%)	5.75	62%
B2	0.18 (3.20%)	0.10 (1.75%)	ND	3.35 (59.3%)	0.92 (16.3%)	5.65	81%
B3	0.22 (3.73%)	0.27 (4.65%)	0.10 (1.62%)	4.17 (70.6%)	1.01 (17.1%)	5.90	98%
B4	0.19 (5.48%)	0.19 (5.68%)	0.06 (1.74%)	2.37 (70.0%)	0.52 (15.3%)	3.38	98%

A1–3, Ambassador variety; B1–4, Premium variety; ND, non-detectable. Recovery expressed as the sum of the Se species (detected by HPLC-ICP-MS vs. total Se determined by ICP-MS).

### Correlations

Significant, strong, and positive correlations between Se dose and seed Se concentration were found for Ambassador from 2014 and for Premium from 2014 and 2015, all with an *r* ^2^ above 0.98 (**Table S1**). For Ambassador from the 2014 growing season, S concentration was significantly and positively correlated with Zn concentration and seed dry matter, while Se concentration was significantly and negatively correlated with number of seeds/pod. For Ambassador from the 2015 growing season, a significant and positive correlation was found between Zn concentration and number of seeds/pod and seed dry matter, between pod length and pod perimeter, and between seed dry matter and pod length. For Premium from the 2014 growing season, a significant and positive correlation was observed between S and Zn concentrations, between Se concentrations and pod perimeter, between number of seeds/pod and pod perimeter and pod length, and between pod perimeter and pod length and seed dry matter. In addition, Zn concentration was significantly and negatively correlated with pod length. For Premium from the 2015 growing season, a significant and positive correlation was found between Se concentration and seed dry matter, and between pod length and pod perimeter, while a significant and negative correlation was found between Zn and Se concentrations (**Table 4**).

**Table 4:** Pearson correlation coefficients between seed mineral concentrations and growth parameters evaluated for two pea varieties (Ambassador and Premium) grown in two seasons (2014 and 2015).

Ambassador variety							
	Se	Zn	S	NS	PP	PL	DM
**Se**		0.397	−0.177	0.208	0.438	−0.048	−0.096
**Zn**	−0.361		−0.046	0.500*	0.039	0.057	0.569**
**S**	−0.219	0.668**		0.227	−0.302	−0.198	0.007
**NS**	−0.566**	0.087	−0.160		−0.258	−0.113	0.302
**PP**	−0.111	0.064	0.232	0.350		0.497*	0.179
**PL**	0.366	−0.158	−0.107	−0.161	0.158		0.524*
**DM**	0.012	0.123	0.494*	−0.270	0.294	0.083	
Premium variety							
	Se	Zn	S	NS	PP	PL	DM
**Se**		−0.609**	−0.382	−0.142	0.185	0.106	0.536*
**Zn**	−0.356		0.301	0.220	0.163	0.302	−0.282
**S**	−0.229	0.706**		−0.283	−0.135	−0.169	0.038
**NS**	0.418	−0.317	0.075		0.182	0.427	−0.354
**PP**	0.617**	−0.308	0.004	0.702**		0.562**	−0.122
**PL**	0.356	−0.485*	0.026	0.757**	0.587**		−0.190
**DM**	0.326	−0.275	−0.387	0.412	0.466*	0.348	

NS, number of seeds per pod; PP, pod perimeter; PL, pod length; DM, seed dry matter. Level of significance, *p < 0.05, **p < 0.01. In light gray—2014 growing season, in dark gray—2015 growing season. In bold—effects consistently significant over two varieties.

## Discussion

In the present study, we investigated the effect of foliar-applied selenate and zinc oxide at the flowering stage on two pea varieties. Parameters of growth; concentrations of Se, Zn, and S; and the species of Se were assessed in seeds. The results highlight that selenate increased seed Se concentration in both pea varieties (**Table 1** and **Figure 1A**) and that the predominant Se species identified in Se-enriched seeds was SeMet (**Table 3** and **Figure S1**). In contrast, zinc oxide had no beneficial effect on seed Zn concentration (**Table 1** and **Figure 1B**). Generally, growth parameters and seed S concentration were not negatively affected by selenate and zinc oxide applications (**Tables 1**, **2**, **Figure 1C**).

Pea was chosen, as it constitutes an important staple legume, with global significance for food security. Previous studies on foliar Se and Zn fertilization indicated that field peas, mainly due to their higher protein concentration, may be more efficient in Se and Zn uptake and seed accumulation than cereals (Poblaciones and Rengel, 2017; Poblaciones and Rengel, 2018). The two pea varieties were selected based on their high-yielding capacity. Selenium and Zn solutions were administered *via* foliar application, as this approach reduced the impact of soil properties on interactions between the examined minerals (Malka et al., 2022). However, differences between soils in 2014 and 2015, as well as climate differences could have contributed to additional variability in Se or Zn foliar uptake. Selenium as selenate was employed due to its accredited high efficiency for foliar uptake (Ros et al., 2016). Though Se is not considered an essential element for higher plants, the beneficial effects of low doses of Se on plant growth, development, and yield, and enhanced resistance to abiotic stresses have been reported, as reviewed previously (Hasanuzzaman et al., 2020). Zinc oxide was tested due to its recommendation by the agro-industry. To the best of our knowledge, there is a research gap regarding effects of foliar-applied zinc oxide on pea uptake. In contrast to Se, Zn is an essential trace element for the plant, influencing crop yield and quality (Hacisalihoglu, 2020).

Neither selenate nor zinc oxide had pronounced effects on growth parameters in our study (except in part for Zn for number of seeds/pod, **Table 2**). Previously tested individual and combined foliar application of sodium selenate and zinc sulfate at early seed filling likewise did not produce differences in the growth of pea (Poblaciones and Rengel, 2017). Similarly, another study did not show any increase in growth parameters of pea upon foliar application of selenate and selenite at the flowering stage (Poblaciones et al., 2013). In contrast, Pandey et al. (2013) found that foliar-applied zinc sulfate at bud initiation had a positive effect on the yield parameters of field pea, including number of flowers, number of pods, their size, and seed numbers. It is possible that differences in Zn status at onset contributed to these observations.

The Premium variety generally accumulated greater amounts of Se in seeds than the Ambassador variety upon biofortification in both years (**Figure 1A**). However, the effect of variety was significant only in the 2015 growing season, despite the fact that seed Se concentration in both varieties was not significantly affected by the growing year. The beneficial effect of foliar-applied selenate on the Se concentration in pea seed is in accordance with previous studies (Smrkolj et al., 2006; Poblaciones et al., 2013; Poblaciones and Rengel, 2017; Poblaciones and Rengel, 2018) and a higher efficiency of foliar-applied selenate than selenite in boosting Se concentration in pea seed was also reported (Poblaciones et al., 2013). The linear and positive trend between foliar selenate treatment and seed Se concentration (**Table S1**) is further in line with previous studies on pea (Poblaciones et al., 2013; Poblaciones and Rengel, 2018).

The health-related effects of Se are expected to mainly depend on the total amount of Se and the chemical speciation of Se in the food crops. Selenium speciation analysis showed that selenomethionine (SeMet) was the predominant Se species identified in seeds of pea treated with 100 g of Se/ha as selenate, with 40%–76% of total Se (**Table 3** and **Figure S1**). A similar proportion of SeMet (49%–67%) was found in seeds of pea upon foliar application of selenate in a previous study (Smrkolj et al., 2006). In the seeds of chickpea treated with foliar Se, a greater proportion of SeMet was obtained in plots fertilized with selenate (84%–91%), followed by those fertilized with selenite (63%–74%) (Poblaciones et al., 2014). A positive effect of foliar (and soil) application of selenate or selenite on the concentrations of organic Se forms (selenocysteine and selenomethionine) was also observed in lentil seeds (Thavarajah et al., 2015). So far, studies on Se fertilization of legumes indicated that Se concentration and speciation in their seeds may be affected by the method of Se application, Se dose, Se form, plant species and variety, and processing (freezing and cooking) (Smrkolj et al., 2006; Poblaciones et al., 2013; Poblaciones et al., 2014; Poblaciones and Rengel, 2017; Poblaciones and Rengel, 2018).

Selenomethionine is especially beneficial for human and animal health, as it is more bioavailable and less toxic than inorganic Se (Schrauzer, 2003; Rayman, 2004; Zhang et al., 2013). Since higher animals and humans are unable to synthesize SeMet in their organs, and the body incorporates it into the protein pool (Schrauzer, 2003), SeMet is a highly suitable form of Se for nutritional supplementation, food fortification, and biofortification. Such strategies may overcome low Se intakes observed in many countries, including European ones (Stoffaneller and Morse, 2015). Selenomethionine and Se-methylselenocysteine exhibit a strong antioxidant activity and have been widely employed as dietary supplements in the chemoprevention of chronic diseases including cancer, diabetes, and cardiovascular diseases (Zhang et al., 2013; Gómez-Jacinto et al., 2020). Kirby et al. (2008) observed that increases in Se plasma concentrations were much higher (∼40%) in a trial group consuming biofortified wheat biscuits that contained a higher SeMet fraction than a group consuming biscuits with a lower proportion of SeMet. In the present study, the high percentage of SeMet in Se-enriched pea seeds suggests that Se in these seeds could be effectively accumulated and transformed into health-promoting Se species.

Unlike Se, our results showed that zinc oxide did not positively affect Zn concentration in pea seeds (**Figure 1B**). However, the beneficial effects of foliar-applied zinc sulfate on Zn concentration in pea seed were reported previously (Pandey et al., 2013). This may indicate that zinc sulfate is a more suitable form of Zn to be employed in further foliar Zn fertilization studies of pea. However, other Zn forms should also be considered. A recent trial on corn showed that Zn, when foliar-applied in complexed form, both as ZnEDTA and especially as glycine-chelated Zn complex (ZnGly), may pose interesting novel candidates to improve Zn accumulation in the plant, with possible differing release kinetics. They also were of lower phytotoxicity than zinc sulfate (Xu et al., 2022), allowing applications at a wider dose range. ZnGly would also be a source of nitrogen.

When investigating effects on S, selenate had no beneficial impact on its concentration in pea seeds (**Figure 1C**). Owing to the chemical similarity between Se and S, the availability of S plays a crucial role in Se accumulation due to competitive effects in their absorption, translocation, and assimilation (Abdalla et al., 2020). It is still not clear whether sulfate transporters in non-hyperaccumulators take up S preferentially over Se. Therefore, it has been proposed that Se and S acquisition can influence one another mutually, which was demonstrated by a significant correlation between Se and S tissue accumulations (Abdalla et al., 2020). In contrast, no significant correlations were found between Se and S concentrations in pea seeds. However, significant and positive correlations were observed between Zn and S concentrations in seeds of two pea varieties for one growing season (**Table 4**), which deserves further investigation, though positive physiological interactions were reported earlier for grains (Cakmak et al., 2010).

In the present study, the significant increase in seed Se accumulation (**Figure 1A**) indicated efficient absorption and mobility of foliar-applied Se. In contrast, non-significant changes in seed Zn accumulation (**Figure 1B**) may suggest low absorption or mobility of foliar-applied Zn. It is worth noting that there is still a knowledge gap on the regulation of Se/Zn transport in the plant following foliar Se/Zn application (Cardini et al., 2021). Deciphering these mechanisms is relevant to improve the efficiency of foliar Se/Zn fertilization. This would also be relevant in sight of a potential co-application of Se and Zn, as it is unclear whether uptake occurs *via* the same mechanisms, e.g., involving similar carriers. Such interactions are important in sight of Se and Zn levels in seeds, and eventually for nutritional aspects. Considering the findings regarding Se intake recommendations, consumption of 100 g of seeds of pea biofortified by 50 and 100 g of Se/ha would be a good source of Se, although it would already exceed the RDA for Se (55 µg, **Table 5**).

**Table 5:** Selenium intake and percentage of recommended dietary allowance for Se (% RDA) covered by 100 g of pea seeds.

Variety/Year	Se treatment(g of Se/ha)	Se intake form 100 g(μg/day)	% RDA from 100 g (USDA*)	% RDA from 100 g (EFSA*)
**Ambassador**				
2014				
	0	8	15	11
	50	259	471	370
	100	487	885	696
2015				
	0	12	22	17
	50	126	229	180
	100	299	544	427
**Premium**				
2014				
	0	16	29	23
	50	428	778	611
	100	784	1,425	1,120
2015				
	0	9	16	13
	50	314	571	449
	100	576	1,047	823

*55 and 70 μg RDA (recommended dietary allowance) and AI (adequate intake) according to USDA and EFSA, respectively.

## Conclusions

In summary, the present study highlighted that selenate and zinc oxide had no marked negative effects on growth parameters of pea varieties, in line with a lack of toxic effects. The lack of beneficial effects of zinc oxide on seed Zn accumulation suggests that future studies on pea should focus on more readily available forms of Zn, such as zinc sulfate. In contrast, selenate substantially improved seed Se accumulation in both varieties with increasing Se dose. However, selenate had no beneficial influence on seed S accumulation, which may suggest no perturbed amino acid regulation in pea.

Small amounts of pea biofortified with 50 g of Se/ha would cover the RDA of Se; however, a very high intake may not be recommended, as the UL could be reached (400 μg). Lower selenate doses could be employed in future studies. Also, evaluating the impact of climate conditions on the investigated parameters warrants further experiments under field conditions, which was not the aim of the present study. Our study highlights the effectiveness of foliar biofortification of pea with Se, which could be a promising strategy to improve human nutrition.

## Data availability statement

The original contributions presented in the study are included in the article/**Supplementary Material**. Further inquiries can be directed to the corresponding author.

## Author contributions

MM designed and carried out the experiments, as well as prepared the first version of the manuscript. TB was involved in the supervision of several analyses, as well as statistical interpretation and manuscript writing. JL carried out analyses of selenium speciation. GL was involved in the conceptualization, supervision of the project and writing of the manuscript. All authors contributed to the article and approved the submitted version.

## Funding

Support for this study was provided by the Ministry of Education, Science, Research and Sport of the Slovak Republic (grant: VEGA 1/0105/14). We thank the staff of the Slovak University of Agriculture in Nitra for helping with the organization of experiments.

## Conflict of interest

The authors declare that the research was conducted in the absence of any commercial or financial relationships that could be construed as a potential conflict of interest.

## Publisher’s note

All claims expressed in this article are solely those of the authors and do not necessarily represent those of their affiliated organizations, or those of the publisher, the editors and the reviewers. Any product that may be evaluated in this article, or claim that may be made by its manufacturer, is not guaranteed or endorsed by the publisher.
