# Supplementary material for: Separate foliar sodium selenate and zinc oxide application enhances Se but not Zn accumulation in pea (Pisum sativum L.) seeds
Source: Front Plant Sci. 2022 Nov 1;13:968324. doi: 10.3389/fpls.2022.968324 (PMC9714566; doi:10.3389/fpls.2022.968324)

Figure S1. Anion exchange HPLC-ICP-MS chromatograms of Se speciation in seeds of Ambassador variety (A1-3) and Premium variety (B1-4) treated with foliar application of 100 g Se/ha as selenate compared with that of a standard solution of 100 µg/L (C) containing five Se species: SeCys, SeMetSeCys, Na_2SeO_3, SeMet and Na_2SeO_4.

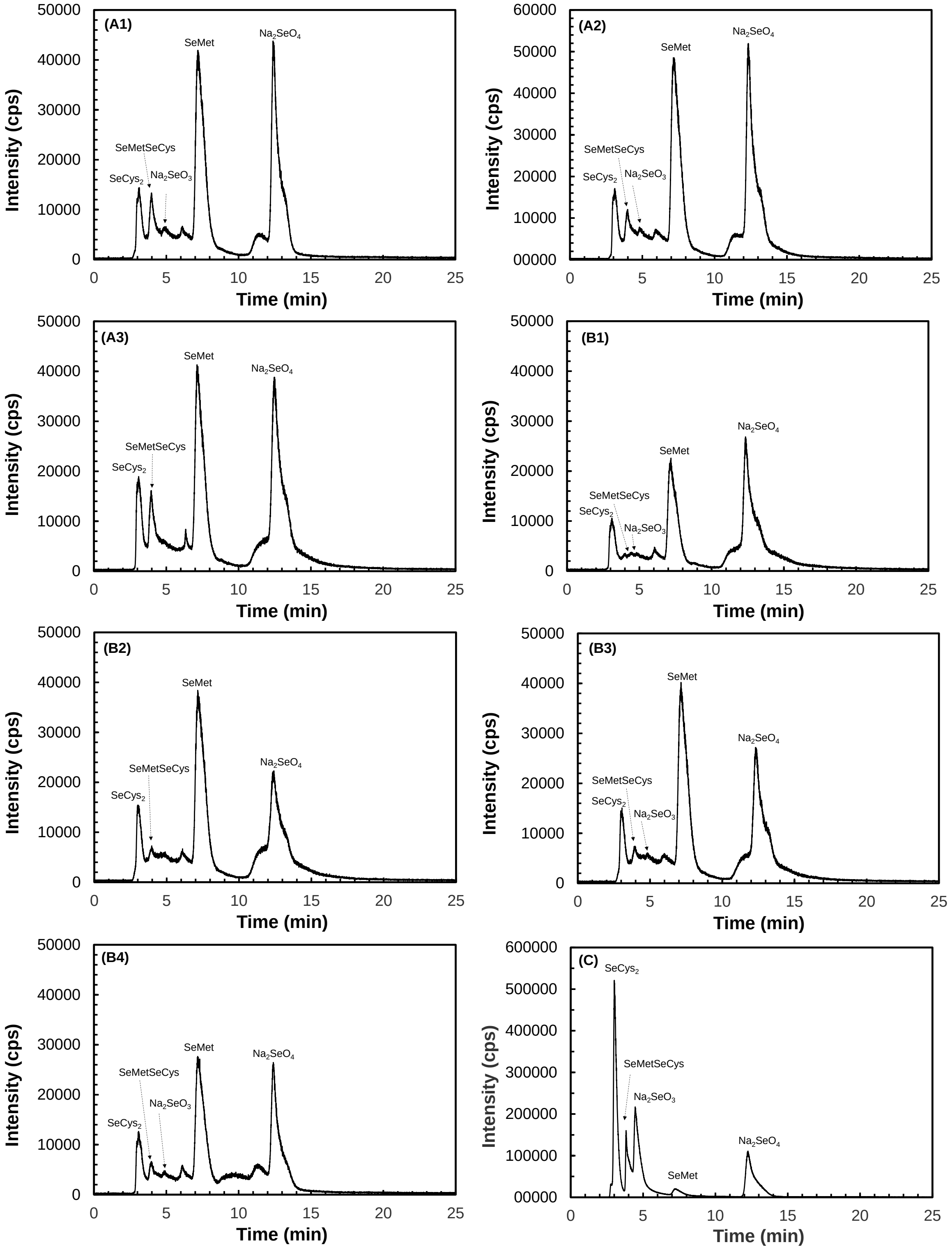

Supplement: Supplementary file 1 [file Image_1.pdf]
