# Supplementary material for: Separate foliar sodium selenate and zinc oxide application enhances Se but not Zn accumulation in pea (Pisum sativum L.) seeds
Source: Front Plant Sci. 2022 Nov 1;13:968324. doi: 10.3389/fpls.2022.968324 (PMC9714566; doi:10.3389/fpls.2022.968324)
Supplement: Supplementary file 2 [file Table_1.docx]

**Table S1.** Correlation analysis between Se dose treatment and seed Se concentration in the pea seed varieties investigated.

| **Year** | **Variety** | **Regression equation** | **r^2^** | **P-value** |
| --- | --- | --- | --- | --- |
| 2014 | Ambassador | y = 0.0479x + 0.1174 | 0.999 | 0.017 |
| 2014 | Premium | y = 0.0768x + 0.2556 | 0.998 | 0.027 |
| 2015 | Ambassador | y = 0.0286x + 0.0246 | 0.986 | 0.075 |
| 2015 | Premium | y = 0.0567x + 0.1645 | 0.998 | 0.029 |
